# Myopericytoma of the thoracic spine in a pregnant woman: a case report on the management of a rare neoplasm

**DOI:** 10.31744/einstein_journal/2025RC1150

**Published:** 2025-08-12

**Authors:** Adriano Jander Ferreira, Túlio Luiz Marra Négri, Julia Hailer Vieira, Anderson Alves Dias, Mellanie Starck Possa, Giovanni Bessa Pereira Lima

**Affiliations:** 1 Universidade Federal do Triângulo Mineiro Hospital de Clínicas Orthopedics and Traumatology Department Uberaba MG Brazil Orthopedics and Traumatology Department, Hospital de Clínicas, Universidade Federal do Triângulo Mineiro, Uberaba, MG, Brazil.; 2 Universidade Federal do Triângulo Mineiro Postgraduate Student in Health Sciences Uberaba MG Brazil Postgraduate Student in Health Sciences, Universidade Federal do Triângulo Mineiro, Uberaba, MG, Brazil.; 3 Universidade Federal do Triângulo Mineiro Hospital de Clínicas Clinical Medicine Unit Uberaba MG Brazil Clinical Medicine Unit, Hospital de Clínicas, Universidade Federal do Triângulo Mineiro, Uberaba, MG, Brazil.; 4 Universidade Federal do Triângulo Mineiro Hospital de Clínicas Radiology and Imaging Diagnosis Department Uberaba MG Brazil Radiology and Imaging Diagnosis Department, Hospital de Clínicas, Universidade Federal do Triângulo Mineiro, Uberaba, MG, Brazil.

**Keywords:** Myopericytoma, Spinal neoplasms, Metastasis neoplasms, Vascular tissue neoplasm, Pregnancy, Radiotherapy

## Abstract

Myopericytomas are perivascular myoid neoplasms that rarely exhibit malignant characteristics. They usually arise in the dermis or subcutaneous tissue and exceptionally involve deep soft tissues, with spinal localization being rare. We report the case of a previously healthy 32-year-old pregnant woman who presented with pain in the interscapular region and progressive loss of lower limb strength. Magnetic resonance imaging revealed a solid neoplastic mass at the T3 level, with spinal canal invasion and spinal cord signal abnormalities. The pregnancy was terminated, followed by lesion embolization, decompressive laminectomy, and local radiotherapy. The patient completely recovered from her neurological symptoms. Thoracic spine myopericytomas are rare and have been poorly reported in the literature. No studies have described the aforementioned neoplasm in a pregnant patient. We report this case because of its peculiarity, evolution, and outcome.

## INTRODUCTION

Myopericytoma is a distinct perivascular myoid neoplasm that shares a morphological spectrum with myofibromas. They rarely exhibit malignant characteristics and arise in the dermis or subcutaneous tissue, occasionally involving the deep soft tissues.^(1)^ Localization in the spine is rare, with only a few cases reported in the literature—the first of which was documented in 2003.^(2-7)^ Specifically, only three cases have been reported in the thoracic spine.^(3,5,7)^ Myopericytomas can affect individuals of any age; however, they are most commonly seen in adults. Mutations in the platelet-derived growth factor receptor beta gene appear to represent a common pathogenesis of myopericytoma.^(1,8)^ Histologically, they are non-encapsulated, well-circumscribed nodular or lobular lesions composed of cytologically bland, oval to fusiform myoid tumor cells, with characteristic multilayered concentric growth around small vessels. Immunohistochemically, myopericytomas express smooth muscle actin and h-caldesmon and are only focally positive for desmin and/or CD34.^(1)^ Because of their rarity, there are few reports on their management, with resection followed by radiotherapy described in two cases.^(3,5)^

## CASE REPORT

**Figure 1:**
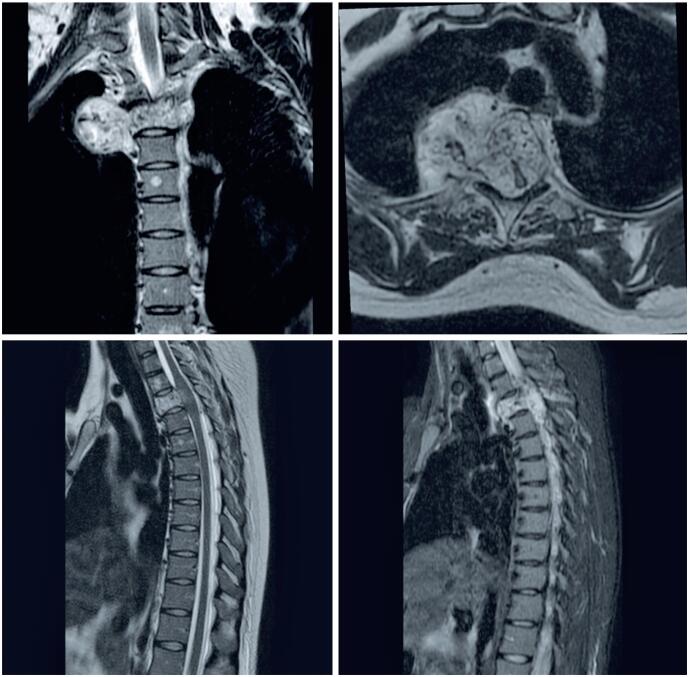
Magnetic resonance imaging of the thoracic spine. Solid neoplastic mass at the T3 level, with diffuse vertebral body infiltration, vertebral canal invasion, and extraosseous paravertebral and foraminal extension, resulting in spinal cord compression

A 32-year-old white woman, who was 34 weeks and five days pregnant and previously healthy, started experiencing pain in the interscapular region associated with progressive weakness in the lower limbs and paresthesia below the xiphoid process. After three weeks, the patient became paraplegic. Physical examination revealed deep reflexes in the lower limbs, cutaneous-plantar reflex in extension, and clonus. Magnetic resonance imaging (MRI) was performed without paramagnetic contrast because of pregnancy. The scan revealed a solid neoplastic mass at the T3 level, with diffuse vertebral body infiltration, spinal canal invasion, and extraosseous paravertebral and foraminal extension, resulting in spinal cord compression (Figure 1).

Because of spinal cord compression and evidence of a neoplastic lesion, corticosteroid therapy was initiated for fetal lung maturation to terminate the pregnancy for subsequent local therapy.

**Figure 2:**
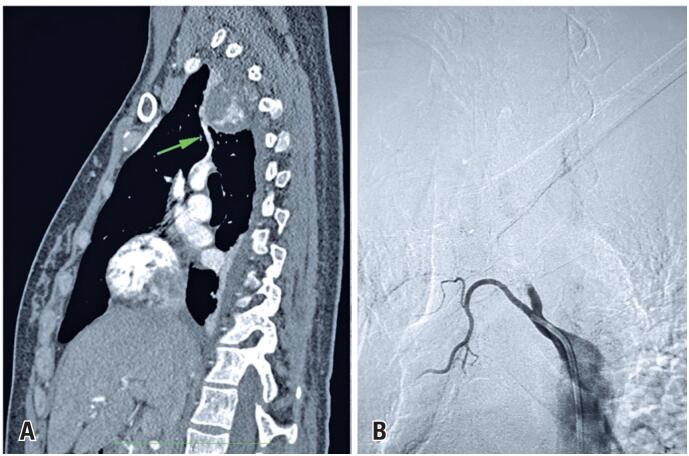
Endovascular embolization. The green arrow on the computed tomography scan demonstrates the vessel nourishing the neoplastic mass (A). After embolization, the flow to the tumor mass is interrupted (B)

The baby was delivered through caesarean section on the 7^th^ day of corticosteroid therapy, and the patient was transferred to our unit on the 5^th^ day of puerperium. The patient underwent staging with computed tomography scans of the chest and abdomen and bone scintigraphy that revealed a single localized skeletal lesion. Subsequently, endovascular embolization of the lesion was performed (Figure 2). On the 4^th^ day of admission, decompressive laminectomy was performed on the 3^rd^ and 4^th^ thoracic vertebrae without instrumentation, and the excised material was sent for anatomopathological examination. By the 2^nd^ postoperative day, the patient exhibited partial enhancement of paresthesia and motor condition (grade 3/5 strength).

**Figure 3:**
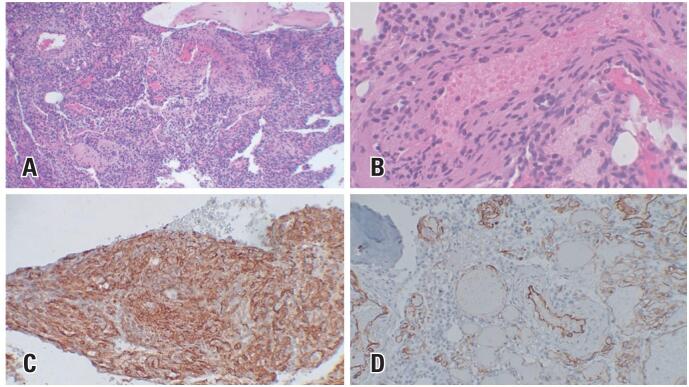
Histology slides. The cancellous bone infiltrated by a histologically benign neoplasm composed of spindle cells and myxoid tissue with abundant capillary blood vessels (A and B). Immunohistochemical analysis revealing positivity for smooth muscle actin markers in the perivascular region (C) and CD34 only in the vessels (D)

Pathological examination revealed a cancellous bone infiltrated by a histologically benign neoplasm composed of spindle cells and myxoid tissue with abundant capillary blood vessels. The immunohistochemical findings were compatible with benign myopericytoma, revealing smooth muscle actin positivity in the perivascular region, CD34 positivity only in the vessels, and negativity in neoplastic cells (Figure 3).

**Figure 4:**
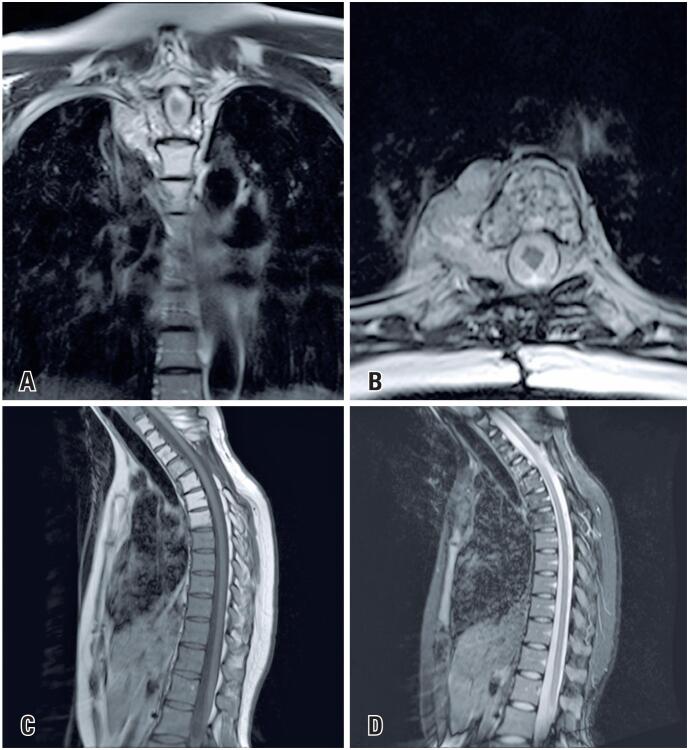
Late magnetic resonance imaging of the thoracic spine. A post-radiotherapy control study with actinic liposubstitution of the vertebral bone marrow and a slight reduction in the solid neoplastic mass at T3 compared with that of the initial examination, and a compressive effect on the spinal cord

The patient underwent local radiotherapy starting 21 days after surgery, with a total dose of 4500 cGy over 25 sessions of 180 cGy each, and started physiotherapy rehabilitation. Her neurological condition progressively enhanced; she regained the ability to walk with support during radiotherapy and was assessed monthly on an outpatient basis. Imaging tests were performed every three months. All MRI scans revealed a solid neoplastic mass at the T3 level—that remained stable in shape, signal, and size, with diffuse infiltration of the vertebral body and posterior elements, resulting in partial collapse. Additionally, altered bone marrow signaling indicating liposubstitution was observed in the vertebral bodies from C7 to T6, related to radiotherapy (Figure 4). At the last assessment, after five years of follow-up, the patient had no motor and/or sensory deficits.

The study was approved by the Research Ethics Committee of *Hospital de Clínicas, Universidade Federal do Triângulo Mineiro* (CAAE: 77650624.9.0000.8667; #6.685.965).

## DISCUSSION

The study presents rare cases of paraplegia during pregnancy, with causes, such as vertebral tuberculosis,^(9,10)^ ruptured arteriovenous malformation,^(11)^ spontaneous spinal epidural hematoma,^(12,13)^ Guillain-Barre syndrome,^(14)^ and neoplasms.^(15-17)^ Among neoplasms, ependymomas,^(15)^ gestational choriocarcinomas,^(16)^ and spinal meningiomas^(17)^ have been identified as causes of paraplegia in pregnant women. In contrast, myopericytoma has not been identified as a potential cause.

Myopericytoma is a rare soft tissue tumor with a predilection for the distal extremities. It is commonly observed in the skin and subcutaneous tissues, with a benign course.^(18)^ It occurs across a wide age range—beginning from the second decade of life, with a consistently reported male predilection. It usually presents as single or multiple subcutaneous nodules on the extremities, with rare cases of multicentricity.^(3)^ Myopericytoma affecting the skeletal system is rare, with only three reported cases affecting the axial skeleton—all located in the thoracic spine at the T3,^(5)^ T5/T6,^(7)^ and T8^(3)^ vertebrae. In this report, we describe the case of a 32-year-old pregnant woman diagnosed with myopericytoma at the T3 level, presenting with neurological symptoms, making this case unique.

In the aforementioned cases, the patients underwent surgery, and tissue samples were sent for histopathological examination. The findings revealed concentric perivascular proliferation of round-to-spindle-shaped cells with myoid differentiation, compatible with the diagnosis of myopericytoma. Immunohistochemistry revealed a pattern consistent with that of myopericytoma.^(3,5,7)^ Therefore, it is a benign tumor; however, rare cases of malignant myopericytoma have been reported.^(19)^

In one of the reported cases, tumor embolization was performed before surgery.^(7)^ In this case, angiotomography revealed a large vessel nourishing the tumor mass; therefore, prior embolization was performed.

In the previously described two cases, radiotherapy was used as an adjuvant to surgical treatment at a total dose of 4500 cGy.^(3,5)^ The current case followed the same postoperative radiotherapy protocol; however, in the previously mentioned cases, the resected tumor volume was considered larger than that of the present case—that underwent only decompressive laminectomy.

In this case, the pregnancy was terminated because of the severity of the neurological symptoms and gestational age, which ensured the safety of the newborn. We emphasize that multidisciplinary discussions are essential, and each case should be approached individually.

Therefore, we conclude that myopericytoma cases of the thoracic spine with compressive symptoms can achieve favorable outcomes, even in peculiar situations, such as those described above, based on the therapy used.
